# Long Sleep Duration and Risk of Ischemic Stroke and Hemorrhagic Stroke: the Kailuan Prospective Study

**DOI:** 10.1038/srep33664

**Published:** 2016-09-16

**Authors:** Qiaofeng Song, Xiaoxue Liu, Wenhua Zhou, Ling Wang, Xiang Zheng, Xizhu Wang, Shouling Wu

**Affiliations:** 1 Department of Cardiology, Tangshan People’s Hospital, North China University of Science and Technology, Tangshan, China; 2 Department of Pneumology, Hospital Affiliated to North China University of Science and Technology, Tangshan, 063000 China; 3 Department of Nuclear Magnetic Resonance Imaging, Tangshan People’s Hospital, North China University of Science and Technology, Tangshan, China; 4 Department of Cardiology, Kailuan Hospital, North China University of Science and Technology, Tangshan, China

## Abstract

The objective of this study was to examine the relationship between sleep duration and ischemic and hemorrhagic stroke in a community-based cohort. The current analysis included 95,023 Chinese participants who were free of stroke at the baseline survey (2006–2007). Cox proportional hazards models were used to calculate hazard ratios (HRs) and their confidence intervals (CIs) for stroke, according to sleep duration. After a mean follow-up period of 7.9 years, 3,135 participants developed stroke (2,504 ischemic stroke and 631 hemorrhagic stroke). The full adjusted hazard ratio (95% CI) of total stroke (with 6–8 hours of night sleep being considered for the reference group) for individuals reporting greater than 8 hours was 1.29 (1.01–1.64). More significant association between long sleep duration and total stroke was found in the elderly (HR, 1.47; 95% CI, 1.05–2.07). Compared with participants getting 6–8 hours of sleep, only women who reported sleeping more than 8 hours per night were associated with hemorrhagic stroke (HR, 3.58; 95% CI, 1.28–10.06). This study suggested that long sleep duration might be a potential predictor/ marker for total stroke, especially in the elderly. And long sleep duration increased the risk of hemorrhagic stroke only in women.

Stroke is one of the leading causes of morbidity and mortality worldwide. More than 7 million Chinese are estimated to experience stroke each year, with 2 million of them being newly diagnosed^1^. Thus, the growing disease burden of stroke points toward prevention as a necessary strategy, which in turn highlights the need for awareness regarding risk factors and warning signs^2^. Recently, sleep has been associated with stroke as most stroke risk factors are modified by sleep and sleep-related disorders^3^,^4^,^5^,^6^,^7^. Further, several studies have consistently demonstrated that individuals with aberrant sleep durations have higher risk of adverse events than individuals reporting more customary sleep durations of 6–8 hours^6^,^7^.

However, evidence is mixed and inconsistent regarding this association between sleep duration and risk of stroke. Some studies have suggested that only long sleep duration (≥10 h or >8 h/d)^3^,^5^,^6^,^7^ is associated with an increased risk of stroke, while others indicate only short sleep duration (<6 h/d) is associated with the higher risk^4^,^8^. In contrast, two recent publications reported that both short and long sleep durations were associated with an increased risk of developing stroke^9^,^10^. Among past studies, only one study has reported an association between sleep patterns and the risk for ischemic stroke in postmenopausal women^5^. Additionally, most studies have not distinguished ischemic stroke from hemorrhagic events, even though there is little neurobiological rationale to suggest that hemorrhagic stroke would be associated with either short or long sleep duration. Hemorrhagic stroke has the highest mortality rate of all stroke subtypes, despite accounting for only 10–15% of all strokes^11^. And there may be age effect on the risk of stroke. Thus, further studies on the relationship linking sleep duration with stroke subtypes stratified by sex and age are required.

To address the aforementioned uncertainty in relating sleep duration to increased risk of stroke, we conducted a longitudinal analysis focusing primarily on ischemic stroke and hemorrhagic stroke, using the comprehensive data from the Kailuan Study.

## Results

At baseline, participants were aged 18 to 98 years (mean 51.2). The percent of participants who reported sleeping for <6 h, 6–8 h, and >8 h per night were 7.0%, 90.3%, and 1.7%, respectively. Table 1 shows baseline characteristics by sleep duration. Participants with >8 h of sleep were more likely to be women. Significant associations were found among sleep duration and age, sex, education level, income level, smoking status, drinking status, physical activity, family history of stroke, body mass index, systolic blood pressure, diastolic blood pressure, fasting blood glucose, total cholesterol, hypotensive drug use, lipid-lowering drug use, hypoglycemic drug use, history of myocardial infarction, snoring status, high sensitive C-reactive protein, and atrial fibrillation.

Table 2 shows the hazard ratios for total stroke according to sleep duration in the total population and stratified by gender. A total of 3,135 participants had at least one stroke incident during the follow-up (mean 7.9 years). Among them, 2,802 were men, and 333 were women. Analysis of the full adjusted data using model 4 indicated that participants in the >8 h group were more likely to develop stroke than those in the 6–8 h group (HR, 1.29; 95% CI, 1.01–1.64). Using model 2, results showed that women who reported sleeping more than 8 h per night were significantly more likely to develop stroke than the participants who reported getting between 6 and 8 h of sleep per night (HR, 2.33; 95% CI, 1.19–4.55). In contrast, men who slept more than 8 h were actually slightly less likely to develop stroke (HR, 1.31; 95% CI, 1.02–1.69). While this association was stronger for women, a formal test for sex difference was not statistically significant (*P* = 0.40).

Table 3 shows the hazard ratios for ischemic stroke according to sleep duration. Among 3,153 incident stroke participants, 2,504 were diagnosed with ischemic stroke. Overall, compared with participants getting 6 to 8 hours of sleep per night, those who reported sleeping more than 8 h were not associated with ischemic stroke (HR, 1.19; 95% CI, 0.90–1.57). No associations were found between long sleep duration and ischemic stroke in women (HR, 1.30; 95% CI, 0.53–3.18) or men (HR, 1.19; 95% CI, 0.90–1.59).

Table 4 shows the hazard ratios for hemorrhagic stroke according to sleep duration. Among 3,153 incident stroke participants, 631 were diagnosed with hemorrhagic stroke. Women who reported sleeping more than 8 h per night were significantly more likely to develop hemorrhagic stroke than those who reported getting 6 to 8 hours of sleep per night (HR, 3.58; 95% CI, 1.28–10.06) (model 4), whereas men who slept more than 8 hours were actually slightly less likely to develop hemorrhagic stroke (HR, 1.38; 95% CI, 0.77–2.45) (model 4). Moreover, the association between long sleep duration and the risk of hemorrhagic stroke remained significant in women even after excluding individuals with myocardial infarction and cancer from the analysis.

Tables 5, 6, 7 summarized hazard ratios (95% CI) for total stroke, ischemic and hemorrhagic stroke according to sleep duration stratified by age, respectively. Analysis of the full adjusted data using model 4 indicated that participants aged more than 60 years in the >8 h group were more likely to develop stroke than those in the 6–8 h group (HR, 1.47; 95% CI, 1.05–2.07) (Table 5). No associations were found between long sleep duration and ischemic or hemorrhagic stroke in participants with different age group (Tables 6 and 7).

## Discussion

We observed an association between long sleep duration and stroke incidence in a large prospective cohort study in China. During a the follow-up period, participants who self-reported sleeping more than 8 hours a night on average had an increased risk for developing stroke compared with those getting 6 to 8 hours of sleep per night. Moreover, age- and sex-stratified analysis showed a marked association of sleep duration with stroke in the elderly and women participants. The association between long sleep duration and stroke was more significant in the elderly. Additionally, our data showed that the association of long sleep duration (>8 h per night) was only associated with hemorrhagic stroke in women. A sensitivity analyses further confirmed these findings.

In accordance with previous studies^3^,^5^,^6^,^10^,^12^, we found a significant association between long sleep and increased total stroke risk. A study from the European Prospective Investigation of Cancer-Norfolk cohort identified prolonged sleep as a potentially useful marker of future stroke risk in an apparently healthy aging population^7^. The Women’s Health Initiative (WHI) study has also shown similar results on the risk of ischemic stroke among postmenopausal women^5^. We extended our analysis to all stroke events in both sexes, and the data suggested a stronger association of long sleep duration and hemorrhagic stroke in women than in men. While another study from China has found short and long sleep duration to both be associated with mortality from ischemic or unspecified stroke^12^, our study suggests that only long sleep durations are associated with greater risk of total stroke. Indeed, although our results are generally consistent with other studies showing associations of long sleep durations with stroke, no such relationships emerged for individuals reporting sleep durations less than 6 hours.

Few studies on this topic have distinguished between ischemic and hemorrhagic strokes^3^,^5^. In the JACC study, long sleep duration was associated with mortality from ischemic stroke in both men and women^3^, while the results for ischemic stroke in our study did not reach statistical significance in the multivariate adjusted model. The WHI study specifically only evaluated the relationship between long sleep duration and ischemic stroke in postmenopausal women. The JACC study found that long sleep duration was only associated with mortality from hemorrhagic stroke in men^3^ in the age-adjusted model, while the results in their study did not reach statistical significance in the multivariate adjusted model. The WHI study did not investigate such a relationship. In complete contrast, the current study found that long sleep duration was only associated with hemorrhagic stroke in women in the multivariate adjusted model. For short sleep duration, results from JACC study were not conclusive^3^. The European Prospective Investigation of Cancer-Norfolk cohort study suggested an association between short sleep duration and risk of ischemic stroke^7^. However, we did not find such a relationship.

Interestingly, our study found an association between long sleep duration and hemorrhagic stroke, which was only significant in women and which remained after patients with myocardial infarction and cancer were excluded from the analysis because their conditions can affect sleep duration. However, we did not find such a relationship in men. The possible reason for differential association of long sleep with stroke between men and women is not fully understood. The differences of hormone secretion, and psychological factors between men and women might account for a differential association of long sleep with stroke. However, the lack of comprehensive information on biological differences between men and women limits us to further investigate whether the relation could be modified or mediated by these factors. Meanwhile, a nationwide, multicenter matched case-control study from Korea suggests that long sleep duration (>8 h) is positively associated with an increased intracerebral hemorrhage risk in a dose-dependent manner^13^. Why long sleep duration is associated with different stroke subtypes is still unclear and more studies are required to replicate our findings.

The underlying mechanism for long sleep duration and stroke risk is not fully understood. One important biological pathway is through inflammation, as long sleep duration has been associated with an increased level of inflammatory biomarkers^14^,^15^. Consistent with above studies, participants with >8 h of sleep have high level of high sensitive C-reactive protein (Table 1). Additionally, epidemiologic evidence has indicated that long sleep duration is related to cardiovascular risk factors or diseases including carotid artery atherosclerosis, atrial fibrillation, white matter hyper-intensity volume, and left ventricular mass, which might predispose one to the risk of stroke^16^,^17^,^18^,^19^,^20^,^21^. In our study, participants with >8 h of sleep were more likely to atrial fibrillation, which was the risk factor of stroke. Previous studies also indicate that longer sleep duration might be reflective of the aging process or periods of failing health and illness^22^,^23^. This evidence further supports the idea that long sleep is a risk factor for stroke.

The strengths of our study include a prospective cohort design, large sample size, Asian ethnicity of the participants, and a broad spectrum of potential confounding parameters. However, potential limitations of our study should also be discussed here. First, we only collected information on sleep duration by self-reported questionnaires at baseline examination. Data of midday nap, excessive daytime sleepiness, sleep quality and sleeping pills use were not undertaken in our study. Thus, nocturnal sleep duration may be different from the whole day sleep duration, especially in China where napping and poor sleep quality are not unusual. And we did not exclude participants with obstructive sleep apnea syndrome (OSAS), which is associated with disrupted sleep/sleep deprivation, excessive daytime sleepiness, and high rates of stroke^24^,^25^,^26^. However, we adjusted snoring status as a confounder in the statistical analysis due to the previous findings on the relationship between habitual snoring and risk of stroke^27^,^28^,^29^. Second, we only investigated the association between sleep duration at baseline examination and future stroke without take change in sleep duration for consideration. Indeed, any subsequent change in sleep duration could lead to non-differential misclassification and potentially underestimate the sleep–stroke association. As a previous study suggested, participants who reported persistently long sleep or substantially increased sleep had a much higher stroke risk^7^. Furthermore, we did not collect sufficient information on the pre- or post-menopause status of women, which appears to be an important determinant of stroke risk in women^5^. Finally, because most of the participants from the Kailuan coal mine were male, the sex distribution of participants was unbalanced, and their health insurance policies were covered by the Kailuan Medical Group. Therefore, they cannot be viewed as a representative sample of the Chinese general population. However, studying such a geographically confined and controlled population greatly reduces residual confounding due to diverse socioeconomic factors and lifestyle patterns.

In summary, this prospective study suggests a significant increase in stroke risk among long sleepers. The underlying neurobiology and mechanistic mediators linking habitual long sleep with increased risk of hemorrhagic stroke need to be investigated. Prolonged sleep might be a useful marker of increased hemorrhagic stroke risk, especially in women, and should be tested further for its utility in clinical practice.

## Methods

### Ethics Statement

The protocol for the study was approved by the Ethics Committee of Kailuan General Hospital in compliance with the Declaration of Helsinki and all participants provided informed written consent with their signatures^30^,^31^.

### Study design and participants

The Kailuan study was a prospective cohort study designed to investigate the association of risk factors and chronic disease. The Kailuan community is located at the center of Kailuan Coal Industry in Hebei Province, China and has approximately 7.2 million inhabitants. There are 11 hospitals responsible for the community’s health care. From June 2006 to October 2007, 155,418 employees (including the retired) in the community were invited to participate, and 101,510 of them (81,110 men, 20,400 women, aged 18–98 years) agreed to enroll in the Kailuan study. After excluding 2,196 participants with self-reported preexisting stroke and 4,129 participants with incomplete sleep data, 66 participants with an average sleep duration at night ≤ 2 hours, and 96 participants with an average sleep duration ≥15 hours, the final study sample included 95,023 participants who had complete data on all covariates. All study participants underwent questionnaire assessment, and clinical and laboratory examination, which were conducted across the 11 hospitals. All doctors and nurses had rigorous unified training before they could conduct the study.

### Assessment of sleep duration

Sleep duration was obtained through a self-reported answer to the question “How many hours of sleep have you had on average night in the preceding 3 months?” Based on the responses, sleep durations were categorized into three groups: short (<6 hours), average (6–8 hours), and long (>8 hours). Additionally, participants were asked to answer “yes” or “no” to the question “Do you generally snore when you sleep?”

### Assessment of potential covariates

All participants underwent a clinical examination and a standardized interview. Physical activity was evaluated based on the responses to questions regarding the types and frequencies of physical activity at work and during leisure time. Physical activity was classified as “≥4 times per week and ≥20 minutes at a time”, “<80 minutes per week”, or “none”. Smoking status and drinking status were classified as “never”, “former”, or “current” according to self-reported information. Family per-member monthly income was categorized as “less than ¥600”, “from ¥600 to ¥799”, “from ¥800 to ¥999”, or “at least ¥1,000”.

Anthropometric parameters such as body height, weight, and waist circumference were measured. Body mass index (BMI) was calculated as kg/m^2^. Systolic blood pressure (SBP) and diastolic blood pressure (DBP) were measured three times in the seated position using a mercury sphygmomanometer, and the average of the three readings was used in the analyses.

Blood samples were collected from the antecubital vein after an overnight fast. All blood samples were tested using a Hitachi 747 auto-analyzer (Hitachi; Tokyo, Japan) at the central laboratory of the Kailuan General Hospital. Fasting blood glucose (FBG) was measured using the hexokinase/glucose-6-phosphate dehydrogenase method^32^.Total cholesterol (TC) was measured using the endpoint test method^33^ (inter-assay coefficient of variation: <10%; Mind Bioengineering Co. Ltd, Shanghai, China). High sensitive C-reactive protein (hs-CRP) was measured by high-sensitivity nephelometry assay (Cias Latex CRP-H, Kanto Chemical, Tokyo, Japan).

### Follow-up and stroke assessment

Participants were followed up by face-to-face interviews at every 2-year routine medical examination until December 31, 2014, or until the event of interest or death. The follow-ups were performed by trained physicians who were blinded to the baseline data. The outcome information was further confirmed by checking discharge summaries from the 11 hospitals and medical records from medical insurance. For the participants without face-to-face follow-ups, outcome information was obtained directly by checking death certificates from provincial vital statistics offices, discharge summaries, and medical records. The primary outcome was the first occurrence of stroke, either the first nonfatal stroke event or stroke death without a preceding nonfatal event. A nonfatal stroke was defined as the sudden onset of a focal neurological deficit with a vascular mechanism lasting more than 24 hours. Cases of fatal stroke were documented by the evidence of a cerebrovascular mechanism. Stroke was diagnosed according to the World Health Organization criteria^34^ combined with brain computed tomography or magnetic resonance imaging for confirmation, and classified into three types: cerebral infarction, cerebral hemorrhage, and subarachnoid hemorrhage. Criteria were consistently applied across all participating hospitals. All stroke outcomes were validated by the Data Safety Monitoring Board and the Arbitration Committee for Clinical Outcomes. Considering the small sample size (n = 44) and different pathogeneses, we did not include the subarachnoid hemorrhage group in this study.

### Statistical analysis

Continuous variables are expressed as means ± standard deviations and categorical variables as percentages. We compared the parameters according to the self-reported sleep duration. One-way analysis of variance (ANOVA) was used for non-paired samples of normally distributed parameters and the Kruskal-Waillis test for non-parametric variables. A chi-squared test was used to compare categorical variables. A multivariate analysis was performed using four models. Model 1 was adjusted for age and sex, and model 2 for age, sex, family per-member monthly income, education level, marital status, smoking status, drinking status, physical activity, and family history of stroke. In addition to the independent parameters analyzed in model 2, a third model included body mass index, systolic blood pressure, diastolic blood pressure, fasting blood glucose, total cholesterol, hypotensive drug use, lipid-lowering drug use, hypoglycemic drug use, history of myocardial infarction, and snoring status. Model4 was stratified by hospitals, and adjusted for the variables in Model 3 plus high sensitive C-reactive protein, and atrial fibrillation. We used Cox proportional-hazards modeling to calculate the hazard ratios (HR) and 95% confidence intervals (CI) of stroke (a group with 6–8-hour sleep duration was used as the reference category). We repeated these analyses for ischemic and hemorrhagic stroke, and present the results by sex and age. Further, we conducted a sensitivity analyses to test the robustness of our findings. Because major fatal diseases (e.g., myocardial infarction and cancer) can impact sleep duration and future stroke risk, we repeated our analysis after excluding individuals with these conditions. The level for statistical significance was set at p ≤ 0.05 (two-tailed). Statistical analysis was performed using SAS 9.3 statistical software (SAS Institute Inc., Cary, NC, USA).

## Additional Information

**How to cite this article**: Song, Q. *et al.* Long Sleep Duration and Risk of Ischemic Stroke and Hemorrhagic Stroke: the Kailuan Prospective Study. *Sci. Rep.* **6**, 33664; doi: 10.1038/srep33664 (2016).

## Figures and Tables

**Table 1:** Baseline characteristics according to sleep duration.

	Sleep duration			
	<6 h	6–8 h	>8 h	*P*
No. of participants	6654	86775	1594	
Male, n(%)	5546 (83.35)	68714 (79.19)	1219 (76.47)	<0.001
Age (years)	55.62 ± 11.89	50.88 ± 12.32	48.86 ± 14.34	<0.001
Marital status (married), %	6066 (91.16)	82205 (94.73)	1429 (89.65)	<0.001
High-school graduate, %	1164 (17.49)	17525 (20.20)	510 (31.99)	<0.001
Family per member monthly income ≥ 800, %	1063 (15.98)	12126 (13.97)	346 (21.71)	<0.001
Physical activity >4 times/week, %	1695 (25.47)	12523 (14.43)	298 (18.70)	<0.001
Current smoker, %	3779 (56.79)	33072 (38.11)	887 (55.65)	<0.001
Current alcohol drinker, %	3802 (57.14)	34097 (39.29)	879 (55.14)	<0.001
Body mass index (kg/m^2^)	24.98 ± 3.48	25.03 ± 3.50	25.08 ± 3.66	<0.001
Systolic blood pressure (mmHg)	133.45 ± 21.62	130.51 ± 20.87	128.59 ± 22.22	<0.001
Diastolic blood pressure (mmHg)	84.08 ± 11.88	83.45 ± 11.76	82.09 ± 12.49	<0.001
Fasting blood glucose (mmol/L)	5.53 ± 1.76	5.47 ± 1.66	5.46 ± 1.68	<0.001
Total cholesterol (mmol/L)	4.99 ± 1.18	4.95 ± 1.15	4.89 ± 1.17	<0.001
High sensitive C-reactive protein, mg/L	0.86 (0.32–2.10)	0.79 (0.30–2.03)	0.89 (0.30–2.17)	<0.001
Atrial fibrillation, %	72 (1.08)	749 (0.86)	21 (1.32)	<0.05
Hypotensive drug use, %	1368 (20.56)	8479 (9.77)	261 (16.37)	<0.001
Lipid-lowering drug use, %	150 (2.25)	649 (0.75)	19 (1.19)	<0.001
Hypoglycemic drug use, %	302 (4.54)	1845 (2.13)	65 (4.08)	<0.001
History of myocardial infarction, %	198 (2.98)	902 (1.04)	28 (1.76)	<0.001
Family history of stroke, %	499 (7.50)	3653 (4.21)	102 (6.40)	<0.001
Snoring status (snored), %	1710 (25.70)	11128 (12.82)	310 (19.45)	<0.001

**Table 2:** Hazard ratios (95% CI) for total stroke according to sleep duration.

	Sleep duration		
	<6 h	6–8 h	>8 h
Cases, (%)	265 (3.98)	2800 (3.23)	70 (4.39)
Model 1†	0.96 (0.85–1.10)	reference	1.37 (1.08–1.74)
Model 2‡	0.96 (0.84–1.09)	reference	1.37 (1.08–1.74)
Model 3§	0.92 (0.81–1.05)	reference	1.29 (1.01–1.64)
Model 4※	0.92 (0.81–1.05)	reference	1.29 (1.01–1.64)
Sensitivity analysis*	0.92 (0.80–1.05)	reference	1.29 (1.01–1.65)
women			
Cases, (%)	32 (2.89)	292 (1.62)	9 (2.40)
Model 1†	1.16 (0.80–1.68)	reference	2.27 (1.16–4.43)
Model 2‡	1.14 (0.79–1.66)	reference	2.33 (1.19–4.55)
Model3§	1.09 (0.75–1.59)	reference	1.91 (0.98–3.74)
Model 4※	1.09 (0.75–1.60)	reference	1.91 (0.98–3.74)
Sensitivity analysis*	1.03 (0.69–1.53)	reference	2.00 (1.02–3.92)
men			
Cases (%)	233 (4.20)	2508 (3.65)	61 (5.00)
Model 1†	0.93 (0.82–1.07)	reference	1.31 (1.02–1.69)
Model 2‡	0.93 (0.81–1.07)	reference	1.31 (1.02–1.69)
Model 3§	0.90 (0.79–1.04)	reference	1.24 (0.96–1.61)
Model 4※	0.90 (0.78–1.03)	reference	1.24 (0.96–1.60)
Sensitivity analysis*	0.91 (0.79–1.04)	reference	1.24 (0.95–1.61)

CI, confidence interval.

^†^Model 1 was stratified by hospitals, and adjusted for age and sex.

^‡^Model 2 was stratified by hospitals, and adjusted for as model 1 plus marital status, family per member monthly income, education level, smoking status, drinking status, physical activity, and family history of stroke.

^§^Model 3 was stratified by hospitals, and adjusted for the variables in Model 2 plus body mass index, systolic blood pressure, diastolic blood pressure, fasting blood glucose, total cholesterol, hypotensive drug use, lipid-lowering drug use, hypoglycemic drug use, history of myocardial infarction, and snoring status.

^※^Model 4 was stratified by hospitals, and adjusted for the variables in Model 3 plus high sensitive C-reactive protein, and atrial fibrillation.

^*^Adjusted for model 3 and further excluded individuals with myocardial infraction and cancer.

**Table 3:** Hazard ratios (95% CI) for ischemic stroke according to sleep duration.

	Sleep duration		
	<6 h	6–8 h	>8 h
Cases (%)	210 (3.16)	2240 (2.58)	54 (3.39)
Model 1†	0.94 (0.82–1.08)	reference	1.30 (0.99–1.70)
Model 2‡	0.92 (0.80–1.07)	reference	1.29 (0.98–1.69)
Model 3§	0.89 (0.77–1.03)	reference	1.20 (0.91–1.57)
Model 4^^※^^	0.89 (0.77–1.03)	reference	1.19 (0.90–1.57)
Sensitivity analysis*	0.89 (0.77–1.04)	reference	1.19 (0.90–1.57)
women			
Cases (%)	25 (2.26)	224 (1.24)	5 (1.33)
Model 1†	1.13 (0.74–1.72)	reference	1.60 (0.66–3.91)
Model 2‡	1.11 (0.72–1.69)	reference	1.64 (0.67–4.00)
Model3§	1.05 (0.68–1.61)	reference	1.31 (0.54–3.21)
Model 4^^※^^	1.05 (0.68–1.61)	reference	1.30 (0.53–3.18)
Sensitivity analysis*	1.01 (0.65–1.58)	reference	1.38 (0.56–3.36)
men			
Cases (%)	185 (3.34)	2016 (2.93)	49 (4.02)
Model 1†	0.91 (0.78–1.06)	reference	1.29 (0.97–1.72)
Model 2‡	0.90 (0.77–1.05)	reference	1.28 (0.97–1.71)
Model 3§	0.88 (0.75–1.02)	reference	1.20 (0.90–1.60)
Model 4※	0.87 (0.74–1.02)	reference	1.19 (0.90–1.59)
Sensitivity analysis*	0.88 (0.75–1.03)	reference	1.18 (0.88–1.59)

CI, confidence interval.

^†^Model 1 was stratified by hospitals, and adjusted for age and sex.

^‡^Model 2 was stratified by hospitals, and adjusted for as model 1 plus marital status, family per member monthly income, education level, smoking status, drinking status, physical activity, and family history of stroke.

^§^Model 3 was stratified by hospitals, and adjusted for the variables in Model 2 plus body mass index, systolic blood pressure, diastolic blood pressure, fasting blood glucose, total cholesterol, hypotensive drug use, lipid-lowering drug use, hypoglycemic drug use, history of myocardial infarction, and snoring status.

^※^Model 4 was stratified by hospitals, and adjusted for the variables in Model 3 plus high sensitive C-reactive protein, and atrial fibrillation.

^*^Adjusted for model 3 and further excluded individuals with myocardial infarction and cancer.

**Table 4:** Hazard ratios (95% CI) for hemorrhagic stroke according to sleep duration.

	Sleep duration		
	<6 h	6–8 h	>8 h
Cases (%)	55 (0.83)	560 (0.65)	16 (1.00)
Model 1†	1.07 (0.81–1.42)	reference	1.64 (0.99–2.69)
Model 2‡	1.10 (0.83–1.45)	reference	1.70 (1.03–2.80)
Model 3§	0.95 (0.73–1.24)	reference	1.60 (0.97–2.63)
Model 4※	1.06 (0.80–1.40)	reference	1.63 (0.99–2.69)
Sensitivity analysis*	1.03 (0.77–1.37)	reference	1.67 (1.01–2.75)
women			
Cases (%)	7 (0.63)	68 (0.38)	4 (1.07)
Model 1†	1.26 (0.57–2.79)	reference	4.49 (1.61–12.49)
Model 2‡	1.29 (0.58–2.88)	reference	4.61 (1.65–12.88)
Model3§	1.01 (0.47–2.15)	reference	3.39 (1.21–9.50)
Model 4※	1.27 (0.57–2.85)	reference	3.58 (1.28–10.06)
Sensitivity analysis*	1.11 (0.47–2.63)	reference	3.60 (1.28–10.12)
men			
Cases (%)	48 (0.87)	492 (0.72)	12 (0.98)
Model 1†	1.05 (0.78–1.41)	reference	1.36 (0.77–2.42)
Model 2‡	1.07 (0.79–1.45)	reference	1.42 (0.80–2.52)
Model 3§	1.05 (0.79–1.40)	reference	1.62 (0.98–2.67)
Model 4※	1.04 (0.77–1.41)	reference	1.38 (0.77–2.45)
Sensitivity analysis*	1.03 (0.75–1.40)	reference	1.41 (0.79–2.50)

CI, confidence interval.

^†^Model 1 was stratified by hospitals, and adjusted for age and sex.

^‡^Model 2 was stratified by hospitals, and adjusted for as model 1 plus marital status, family per member monthly income, education level, smoking status, drinking status, physical activity, and family history of stroke.

^§^Model 3 was stratified by hospitals, and adjusted for the variables in Model 2 plus body mass index, systolic blood pressure, diastolic blood pressure, fasting blood glucose, total cholesterol, hypotensive drug use, lipid-lowering drug use, hypoglycemic drug use, history of myocardial infarction, and snoring status.

^※^Model 4 was stratified by hospitals, and adjusted for the variables in Model 3 plus high sensitive C-reactive protein, and atrial fibrillation.

^*^Adjusted for model 3 and further excluded individuals with myocardial infarction and cancer.

**Table 5:** Hazard ratios (95% CI) for total stroke according to sleep duration stratified by age.

	Sleep duration		
	<6 h	6–8 h	>8 h
Age ≥ 60 years			
Cases (%)	139 (6.44)	1199 (6.75)	36 (11.29)
Model 1†	0.92 (0.77–1.10)	reference	1.59 (1.14–2.22)
Model 2‡	0.92 (0.77–1.11)	reference	1.60 (1.15–2.24)
Model 3§	0.94 (0.78–1.12)	reference	1.49 (1.06–2.09)
Model 4※	0.94 (0.78–1.12)	reference	1.47 (1.05–2.07)
Age < 60 year			
Cases (%)	126 (2.80)	1601 (2.32)	34 (2.67)
Model 1†	1.04 (0.87–1.25)	reference	1.32 (0.94–1.85)
Model 2‡	1.02 (0.85–1.22)	reference	1.28 (0.91–1.81)
Model 3§	0.93 (0.78–1.12)	reference	1.20 (0.86–1.69)
Model 4※	0.93 (0.77–1.12)	reference	1.21 (0.86–1.70)

CI, confidence interval.

^†^Model 1 was stratified by hospitals, and adjusted for age and sex.

^‡^Model 2 was stratified by hospitals, and adjusted for as model 1 plus marital status, family per member monthly income, education level, smoking status, drinking status, physical activity, and family history of stroke.

^§^Model 3 was stratified by hospitals, and adjusted for the variables in Model 2 plus body mass index, systolic blood pressure, diastolic blood pressure, fasting blood glucose, total cholesterol, hypotensive drug use, lipid-lowering drug use, hypoglycemic drug use, history of myocardial infarction, and snoring status.

^※^Model 4 was stratified by hospitals, and adjusted for the variables in Model 3 plus high sensitive C-reactive protein, and atrial fibrillation.

**Table 6:** Hazard ratios (95% CI) for ischemic stroke according to sleep duration stratified by age.

	Sleep duration		
	<6 h	6–8 h	>8 h
Age ≥ 60 years			
Cases (%)	114 (5.29)	982 (5.53)	28 (8.78)
Model 1†	0.93 (0.76–1.13)	reference	1.51 (1.04–2.20)
Model 2‡	0.93 (0.76–1.13)	reference	1.52 (1.04–2.22)
Model3§	0.94 (0.77–1.14)	reference	1.38 (0.94–2.03)
Model 4※	0.94 (0.77–1.14)	reference	1.36 (0.93–2.00)
Age < 60 year			
Cases (%)	96 (2.13)	1258 (1.82)	26 (2.04)
Model 1†	0.99 (0.80–1.22)	reference	1.25 (0.85–1.85)
Model 2‡	0.95 (0.77–1.17)	reference	1.20 (0.82–1.78)
Model 3§	0.88 (0.71–1.09)	reference	1.13 (0.76–1.67)
Model 4※	0.87 (0.70–1.08)	reference	1.13 (0.76–1.67)

CI, confidence interval.

^†^Model 1 was stratified by hospitals, and adjusted for age and sex.

^‡^Model 2 was stratified by hospitals, and adjusted for as model 1 plus marital status, family per member monthly income, education level, smoking status, drinking status, physical activity, and family history of stroke.

^§^Model 3 was stratified by hospitals, and adjusted for the variables in Model 2 plus body mass index, systolic blood pressure, diastolic blood pressure, fasting blood glucose, total cholesterol, hypotensive drug use, lipid-lowering drug use, hypoglycemic drug use, history of myocardial infarction, and snoring status.

^※^Model 4 was stratified by hospitals, and adjusted for the variables in Model 3 plus high sensitive C-reactive protein, and atrial fibrillation.

**Table 7:** Hazard ratios (95% CI) for hemorrhagic stroke according to sleep duration stratified by age.

	Sleep duration		
	<6 h	6–8 h	>8 h
Age ≥ 60 years			
Cases (%)	25 (1.16)	217 (1.22)	8 (2.51)
Model 1†	0.90 (0.60–1.37)	reference	1.89 (0.93–3.83)
Model 2‡	0.91 (0.60–1.38)	reference	1.91 (0.93–3.87)
Model3§	0.94 (0.62–1.44)	reference	1.91 (0.94–3.90)
Model 4※	0.94 (0.62–1.43)	reference	1.90 (0.93–3.88)
Age < 60 year			
Cases (%)	30 (0.67)	343 (0.50)	8 (0.63)
Model 1†	1.27 (0.87–1.84)	reference	1.55 (0.77–3.14)
Model 2‡	1.30 (0.89–1.89)	reference	1.62 (0.80–3.28)
Model 3§	1.16 (0.79–1.70)	reference	1.43 (0.71–2.89)
Model 4※	1.17 (0.80–1.71)	reference	1.45 (0.72–2.93)

CI, confidence interval.

^†^Model 1 was stratified by hospitals, and adjusted for age and sex.

^‡^Model 2 was stratified by hospitals, and adjusted for as model 1 plus marital status, family per member monthly income, education level, smoking status, drinking status, physical activity, and family history of stroke.

^§^Model 3 was stratified by hospitals, and adjusted for the variables in Model 2 plus body mass index, systolic blood pressure, diastolic blood pressure, fasting blood glucose, total cholesterol, hypotensive drug use, lipid-lowering drug use, hypoglycemic drug use, history of myocardial infarction, and snoring status.

^※^Model 4 was stratified by hospitals, and adjusted for the variables in Model 3 plus high sensitive C-reactive protein, and atrial fibrillation.
